# Effects of Maternal Resveratrol on Maternal High-Fat Diet/Obesity with or without Postnatal High-Fat Diet

**DOI:** 10.3390/ijms21103428

**Published:** 2020-05-12

**Authors:** Mei-Hsin Hsu, Jiunn-Ming Sheen, I-Chun Lin, Hong-Ren Yu, Mao-Meng Tiao, You-Lin Tain, Li-Tung Huang

**Affiliations:** 1 Department of Pediatrics, Kaohsiung Chang Gung Memorial Hospital and Chang Gung University College of Medicine, Kaohsiung 833, Taiwan; a03peggy@yahoo.com.tw (M.-H.H.); ray.sheen@gmail.com (J.-M.S.); uc22@adm.cgmh.org.tw (I.-C.L.); yuu2004taiwan@yahoo.com.tw (H.-R.Y.); tmm@adm.cgmh.org.tw (M.-M.T.); tainyl@hotmail.com (Y.-L.T.); 2 Department of Medicine, Chang Gung University, Linkou 333, Taiwan

**Keywords:** maternal high-fat diet, maternal obesity, maternal resveratrol, postnatal high-fat diet, spatial, hippocampus

## Abstract

To examine the effects of maternal resveratrol in rats borne to dams with gestational high-fat diet (HFD)/obesity with or without postnatal high-fat diet. We first tested the effects of maternal resveratrol intake on placenta and male fetus brain in rats borne to dams with gestational HFD/obesity. Then, we assessed the possible priming effect of a subsequent insult, male offspring were weaned onto either a rat chow or a HFD. Spatial learning and memory were assessed by Morris water maze test. Blood pressure and peripheral insulin resistance were examined. Maternal HFD/obesity decreased adiponectin, phosphorylation alpha serine/threonine-protein kinase (pAKT), sirtuin 1 (SIRT1), and brain-derived neurotrophic factor (BDNF) in rat placenta, male fetal brain, and adult male offspring dorsal hippocampus. Maternal resveratrol treatment restored adiponectin, pAKT, and BDNF in fetal brain. It also reduced body weight, peripheral insulin resistance, increased blood pressure, and alleviated cognitive impairment in adult male offspring with combined maternal HFD and postnatal HFD. Maternal resveratrol treatment restored hippocampal pAKT and BDNF in rats with combined maternal HFD and postnatal HFD in adult male offspring dorsal hippocampus. Maternal resveratrol intake protects the fetal brain in the context of maternal HFD/obesity. It effectively reduced the synergistic effects of maternal HFD/obesity and postnatal HFD on metabolic disturbances and cognitive impairment in adult male offspring. Our data suggest that maternal resveratrol intake may serve as an effective therapeutic strategy in the context of maternal HFD/obesity.

## 1. Introduction

Maternal obesity is a global problem, with over 30% of women at child-bearing age being categorized as obese [1]. Obesity in pregnancy is associated with an increased risk of pre-eclampsia, gestational diabetes, miscarriage, premature delivery, and high Cesarean section rate [2]. It is estimated that the economic cost of obesity in pregnancy is greater than USD 100 million annually in the USA [3]. Maternal obesity can cause a cyclical transgenerational transmission of obesity [4].

Epidemiological and experimental studies provide evidence of the long-term deleterious effect of maternal obesity on offspring—the so-called hypothesis of intrauterine programming [5]. Mounting evidence suggests that an intrauterine high-fat environment plays a crucial role in the development of diseases in offspring, such as hypertension, diabetes, and nervous system diseases [6]. Furthermore, maternal high-fat diet (HFD)/obesity may predispose offspring to a subsequent metabolic challenge, and increase the risk of metabolic syndrome in the offspring [7,8,9,10].

Resveratrol (trans-3,5,4′-trihydroxystilbene) is a phenolic compound with a potent antioxidant activity, which is found in various plants such as grapes and berries [11,12]. Resveratrol has a variety of biological and pharmacological effects, including cardioprotection, antioxidant, and anti-inflammatory effects [13,14,15]. It is also known that resveratrol can halt the progression of type 2 diabetes mellitus and metabolic syndrome [16]. Resveratrol has been shown to have beneficial effects against HFD-induced obesity in animal studies [17,18,19], and exerts health benefits to obese individuals [20].

Reprogramming strategies means maneuvers to reverse the malprogrammed development and to resume normal development [19,21,22]. Since maternal obesity can have adverse influences in offspring beginning from embryo development, the potential usefulness of maternal resveratrol administration in reprogramming is worth investigating [11]. Recently, the potential use of resveratrol in adverse human pregnancies is under scrutiny [12].

Thus, the purpose of this study was to investigate whether maternal resveratrol intake was able to reduce placenta and fetus brain insults in rats borne to dams with gestational HFD and obesity. In addition, we examined whether maternal resveratrol was effective in reducing the synergistic effects of maternal HFD/obesity and postnatal HFD on metabolic disturbances and cognitive impairment in adult male offspring.

## 2. Results

### 2.1. Maternal HFD/Obesity Affects Placenta and the Effects of Maternal Resveratrol

**Figure 1:**
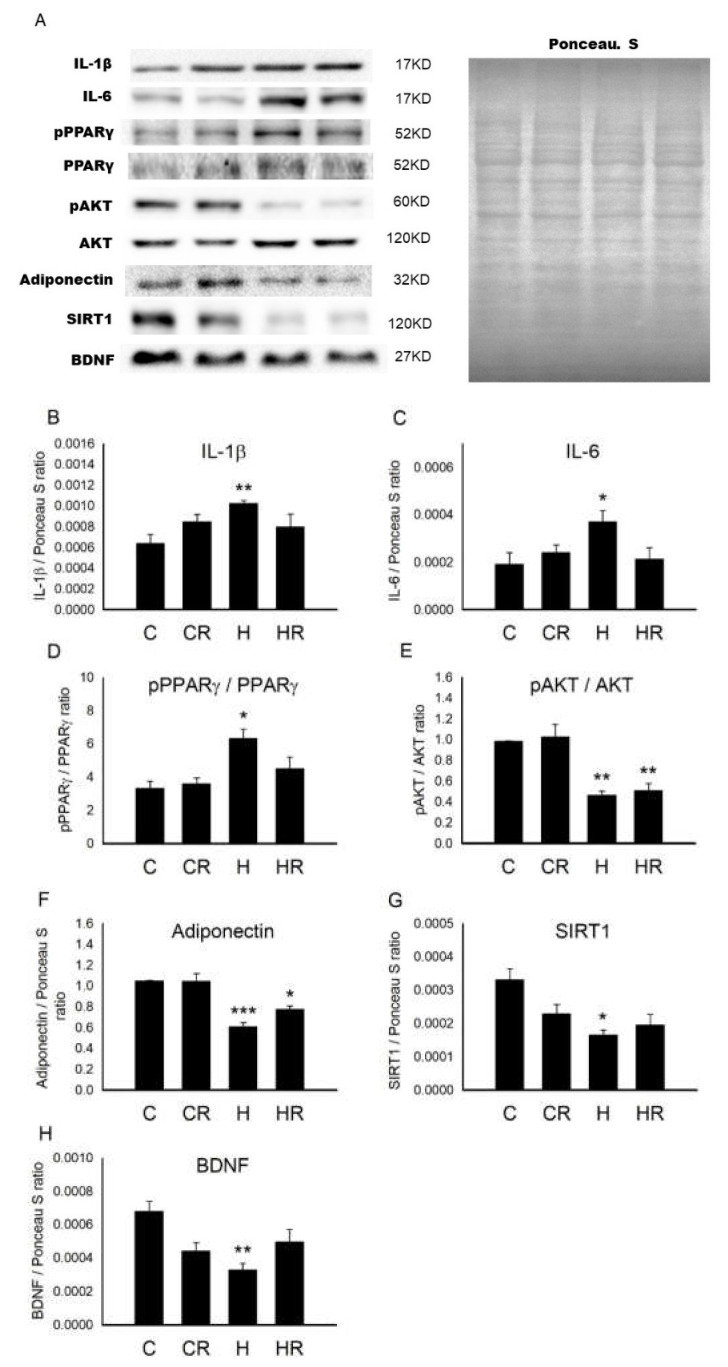
Targeted protein levels in rat placenta. The levels of placenta targeted proteins were detected via Western blotting and normalized using Ponceau S staining. (**A**) Representative band densities are shown. Relative abundance of (**B**) IL-1β, (**C**) IL-6, (**D**) pPPARγ/PPARγ, (**E**) pAKT/AKT, (**F**) adiponectin, (**G**) SIRT1, and (**H**) BDNF were quantified. One-way ANOVA followed by Fisher’s LSD post hoc was used for comparisons among multiple groups. IL-1β: (F (3,16) = 3.390, *p* < 0.05). IL-6: (F (3,24) = 3.673, *p* < 0.05). pPPARγ/PPARγ: (F (3,24) = 3.182, *p* < 0.05). pAKT/AKT: (F (3,20) = 7.166, *p* < 0.01). Adiponectin: (F (3,20) = 9.245, *p* < 0.001). SIRT1: (F (3,24) = 3.224, *p* < 0.05). BDNF: (F (3,24) = 3.288, *p* < 0.05). *n* = 10–12 for each group. * *p* < 0.05 vs. C; ** *p* < 0.01 vs. C; *** *p* < 0.001 vs. C. All data are presented as mean ± SEM. IL-1β; interleukin 1β; IL-6: interleukin-6; PPARγ: peroxisome proliferator-activated receptors γ; AKT: alpha serine/threonine-protein kinase; SIRT1: sirtuin 1; BDNF: brain-derived neurotrophic factor.

Figure 1A showed the representative blots of protein involved in placentation. Maternal obesity results in placental inflammation and increased cytokine production [19]. We first examined the main cytokines during placentation. One-way ANOVA showed a significant difference in interleukin 1 beta (IL-1β) levels among the four groups (*p* < 0.05). Post hoc analysis showed that the maternal HFD/obesity (H group) had higher concentration of IL-1β than the maternal rat chow (C group) (*p* < 0.05); however, there was no significant difference between the H and maternal HFD/obesity + maternal resveratrol (HR groups) (*p* > 0.05) (Figure 1B). Similarly, one-way ANOVA showed a significant difference in interleukin 6 (IL-6) levels among the four groups (*p* < 0.05). Post hoc analysis showed that the H group had higher concentration of IL-6 than the C group (*p* < 0.05); however, there was no significant difference between the H and HR groups (*p* > 0.05) (Figure 1C).

Peroxisome proliferator-activated receptors γ (PPARγ) is the main modulator of mammalian placentation [23]. One-way ANOVA showed a significant difference in pPPARγ/PPARγ levels among the four groups (*p* < 0.05). Post hoc analysis showed that the H group had higher concentration of pPPARγ/PPARγ than the C group (*p* < 0.05); however, there was no significant difference between the H and HR groups (*p* > 0.05) (Figure 1D). AKT is involved in insulin signaling and is altered in obesity status [24]. One-way ANOVA showed a significant difference in pAKT/AKT levels among the four groups (*p* < 0.01). Post hoc analysis showed that the H group had lower concentration of pAKT/AKT than the C group (*p* < 0.01), however, there was no significant difference between the H and HR groups (*p* > 0.05) (Figure 1E). Low maternal adiponectin is implicated in maternal obesity [25]. One-way ANOVA showed a significant difference in adiponectin levels among the four groups (*p* < 0.001). Post hoc analysis showed that the H group had lower concentration of adiponectin than the C group (*p* < 0.001); however, there was no significant difference between the H and HR groups (*p* > 0.05) (Figure 1F). Low placental SIRT1 is noted in maternal obesity [26]. One-way ANOVA showed a significant difference in SIRT1 levels among the four groups (*p* < 0.05). Post hoc analysis showed that the H group had lower concentration of SIRT1 than the C group (*p* < 0.05), however, there was no significant difference between the H and HR groups (*p* > 0.05) (Figure 1G). Maternal BDNF expression is altered in the context of maternal obesity and adverse pregnancy [27]. One-way ANOVA showed a significant difference in BDNF levels among the four groups (*p* < 0.05). Post hoc analysis showed that the H group had lower concentration of BDNF than the C group (*p* < 0.001); however, there was no significant difference between H and HR groups (*p* > 0.05) (Figure 1H). Collectively, maternal HFD/obesity decreased adiponectin, pAKT, SIRT1, and BDNF in rat placenta.

### 2.2. Maternal HFD/Obesity Affects Fetal Brain and the Effects of Maternal Resveratrol

**Figure 2:**
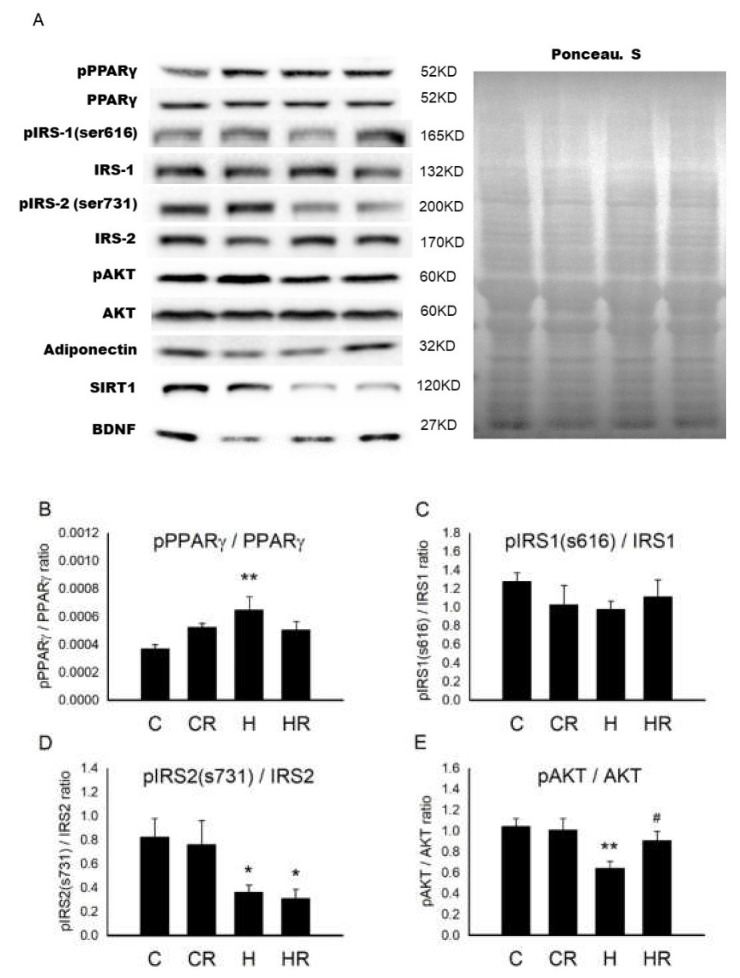

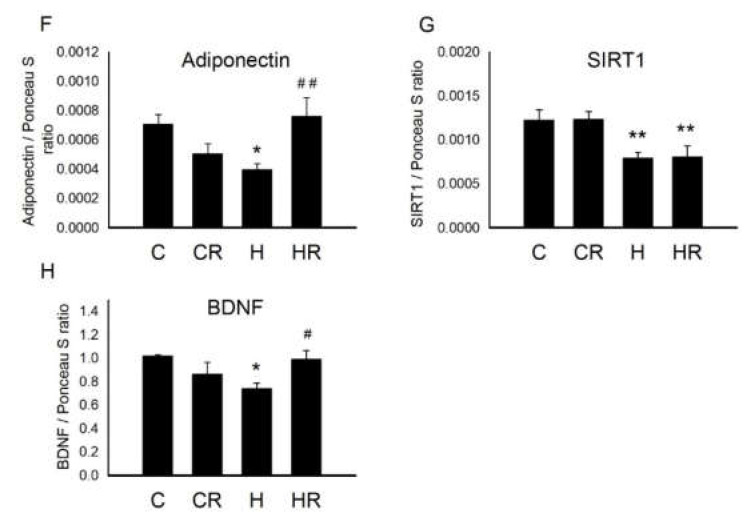
Targeted protein levels in rat fetal brain. The levels of fetal brain targeted proteins were detected via Western blotting and normalized using Ponceau S staining. (**A**) Representative band densities are shown. Relative abundance of (**B**) pPPARγ/PPARγ, (**C**) pIRS1 (s616)/IRS1, (**D**) pIRS2 (s731)/IRS2, (**E**) pAKT/AKT, (**F**) adiponectin, (**G**) SIRT1, and (**H**) BDNF were quantified. One-way ANOVA followed by Fisher’s LSD post hoc was used to assess the significant differences among groups and multiple comparisons. pPPARγ/PPARγ: (F (3,20) = 3.470, *p* < 0.05). pIRS2 (s731)/IRS2: (F (3,24) = 3.647, *p* < 0.05). pAKT/AKT: (F (3,20) = 4.261, *p* < 0.05). Adiponectin: (F (3,20) = 4.226, *p* < 0.05). SIRT1: (F (3,20) = 5.828, *p* < 0.01). BDNF: (F (3,20) = 3.445, *p* < 0.05). *n* = 10–12 for each group. * *p* < 0.05 vs. C; ** *p* < 0.01 vs. C; # *p* < 0.05 vs. H; ## *p* < 0.01 vs. H; PPARγ: peroxisome proliferator-activated receptors γ; IRS: insulin receptor substrate; AKT: alpha serine/threonine-protein kinase; SIRT1: sirtuin 1; BDNF: brain-derived neurotrophic factor.

Figure 2A showed the representative blots of proteins involved in cognition and insulin signaling examined in fetal brain. In fetal brain, one-way ANOVA showed a significant difference in pPPARγ/PPARγ levels among the four groups (*p* < 0.05). Post hoc analysis showed that the H group had higher concentration of pPPARγ/PPARγ than the C group (*p* < 0.01), however, there was no significant difference between the H and HR groups (*p* > 0.05) (Figure 2B). Regarding insulin receptor substrate (IRS)1, there was no significant difference in pIRS1 (s616)/IRS1 (s616) among the four groups (*p* > 0.05) (Figure 2C). However, one-way ANOVA showed decreased pIRS2 (s731)/IRS2 (s731) levels among the four groups (*p* < 0.05). Post hoc analysis showed that the H group had lower pIRS2 (s731)/IRS2(s731) than the C group (*p* < 0.05); however, there was no significant difference between the H and HR groups (*p* > 0.05) (Figure 2D). One-way ANOVA showed a significant difference in pAKT/AKT levels among the four groups (*p* < 0.05). Post hoc analysis showed that the H group had lower pAKT/AKT than the C group (*p* < 0.01). In addition, the HR group had higher pAKT/AKT level than the H group (*p* < 0.05) (Figure 2E). One-way ANOVA showed a significant difference in adiponectin levels among the four groups (*p* < 0.05). Post hoc analysis showed that the H group had lower adiponectin than the C group (*p* < 0.05). In addition, the HR group had higher adiponectin levels than the H group (*p* < 0.01) (Figure 2F). One-way ANOVA showed a significant difference in SIRT1 levels among the four groups (*p* < 0.01). Post hoc analysis showed that the H group had lower SIRT1 than the C group (*p* < 0.01); however, there was no significant difference between the H and HR groups (*p* > 0.05) (Figure 2G). One-way ANOVA showed a significant difference in BDNF levels among the four groups (*p* < 0.05). Post hoc analysis showed that the H group had lower BDNF than the C group (*p* < 0.05). In addition, the HR group had higher BDNF levels than the H group (*p* < 0.05) (Figure 2H). Collectively, maternal HFD/obesity decreased adiponectin, pAKT, SIRT1, and BDNF in male rat fetal brain and maternal resveratrol treatment could restore adiponectin, pAKT, and BDNF.

### 2.3. Body Weights after Birth

**Figure 3:**
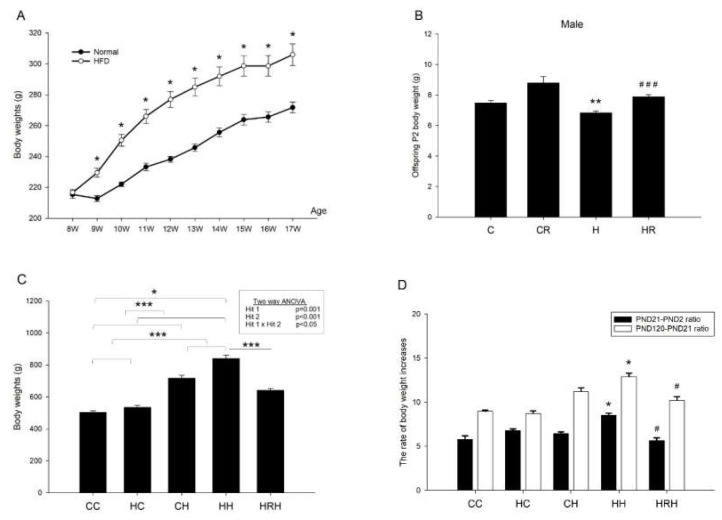
Body weight. (**A**) Female rats were fed a chow diet or a high-fat diet (HFD) and weighed weekly for 8 weeks before mating. *n* = 10–12 for each group. Student’s *t*-test, * *p* < 0.05 vs. HFD; (**B**) After mating, male pups were weighed on PND 2. F (3,92) = 16.075, *p* < 0.0001. *n* = 10–12 for each group. ** *p* < 0.01 vs. C; ### *p* < 0.001 vs. H. One-way ANOVA followed by Fisher’s LSD post hoc was used to assess the significant differences among groups and multiple comparisons. (**C**) The offspring were weaned at 21 days of age and allocated to either the chow diet or HFD ad libitum from weaning up to ~4 months of age. The male rats were weighed at ~4 months old. Body weights were analyzed using two-way ANOVA (maternal diet x post-weaning diet). The therapeutic effect of resveratrol was evaluated using the Student *t*-test. Maternal HFD/obesity: (F (1,53) = 12.131, *p* = 0.001). Postnatal HFD: (F (1,53) = 122.205, *p* < 0.001). An interaction effect between maternal HFD/obesity and postnatal HFD: (F (1,53) = 4.768, *p* < 0.05). Hit 1 indicated CC and CH vs. HC and HH. Hit 2 indicated CC and HC vs. CH and HH. * *p* < 0.05; *** *p* < 0.001. (**D**) At PND 21, the ratio of body weight increases was highest in the HH group (compared with PND 2; * HH vs. CC, CH, and HC, all *p* < 0.05; # HH vs. HRH; *p* < 0.05). At ~PND 120, the ratio of body weight increases was highest in the HH group (compared with PND 21; * HH vs. CC, CH, and HC, all *p* < 0.05; # HH vs. HRH; *p* < 0.05). *n* = 10–12 for each group. HFD: high-fat diet; PND: postnatal day.

Before mating, female rats were fed a chow diet or a HFD and weighed weekly for 8 weeks before mating. HFD-fed dams had higher body weight than the control dams beginning one week after diet through conception (Figure 3A). At the end of gestation, resveratrol did not have an effect on dams’ weight (H vs. HR; 393.84 ± 16.74 gm vs. 390.57 ± 12.7 gm; p > 0.05). One-way ANOVA showed significant differences in weight on postnatal day (PND)2 among the four groups (*p* < 0.0001). Post hoc analysis showed that the H group had lower body weight than the C group (7.48 ± 0.17 gm vs. 6.84 ± 0.12 gm; *p* < 0.01). In addition, the HR group recovered the weight loss in HFD, and there was significant difference in body weight between the H and HR groups (*p* < 0.0001) (Figure 3B). Analysis of weights taken when offspring were 4 months old, showed a significant main effect of both maternal HFD/obesity (*p* = 0.001), and postnatal HFD treatment (*p* < 0.001). There was also a significant interaction effect between maternal HFD/obesity and postnatal HFD (*p* < 0.05). We found adult offspring in the maternal/HFD/obesity + postnatal HFD (HH group) were the heaviest, followed by offspring of the maternal rat chow diet + postnatal HFD (CH group). Student’s *t*-test showed a significant effect of maternal resveratrol treatment on decreased body weights in animals exposed to combined treatments (HH vs. HRH; *p* < 0.001) (HRH: maternal HFD/obesity + maternal resveratrol + postnatal HFD) (Figure 3C). In addition, the ratio of body weight increases was highest in the HH group and resveratrol could decrease the ratio of increase both at PND 21 (HH vs. CC, CH, and HH, all *p* < 0.05; HH vs. HRH; *p* < 0.05) and at ~PND 120 (HH vs. CC, CH, and HH, all *p* < 0.05; HH vs. HRH; *p* < 0.05) (CC: maternal rat chow diet + postnatal chow diet; HC: maternal HFD/obesity + postnatal chow diet) (Figure 3D).

### 2.4. Blood Pressure

**Figure 4:**
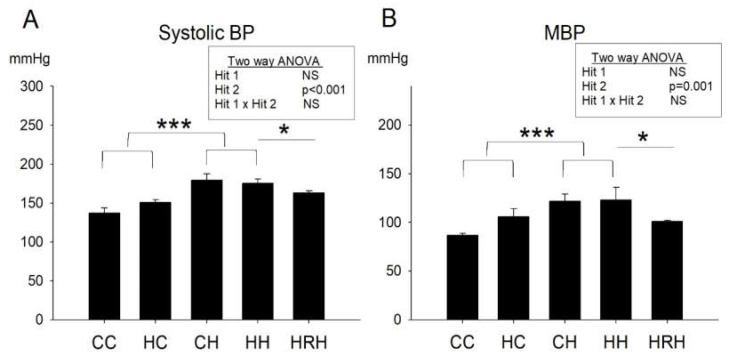
Blood pressures. (**A**) Systolic and (**B**) mean blood pressures of ~4-month-old male rats were analyzed using two-way ANOVA and the therapeutic effect of resveratrol was further evaluated using the Student’s *t*-test. Postnatal HFD treatment on systolic blood pressures: (F (1,14) = 26.396, *p* < 0.001). Postnatal HFD treatment on MBP: (F (1,14) = 16.396, *p* = 0.001). * *p* < 0.05; *** *p* < 0.001. BP: blood pressure; MBP: mean blood pressure.

The blood pressure of 4-month-old male rats was measured. In line with our previous report [9], we observed a significant main effect of the postnatal HFD treatment (*p* < 0.001), however, there was no significant effect of maternal HFD/obesity on systolic blood pressure. Further analysis showed no significant interaction between maternal HFD/obesity and postnatal HFD. This result indicated that postnatal HFD led to increased systolic blood pressure. Additionally, the Student’s *t*-test analysis showed that resveratrol treatment significantly reduced increased systolic blood pressure levels in animals with 2 hits (HRH vs. HH; *p* < 0.05) (Figure 4A). In terms of mean arterial pressure, we observed a main effect for the postnatal HFD treatment (*p* = 0.001); however, maternal HFD/obesity did not significantly affect mean arterial pressure. Two-way ANOVA showed no significant interaction between maternal HFD/obesity and postnatal HFD. Further analysis using the Student’s *t*-test showed that resveratrol treatment significantly reduced mean arterial pressure in animals with 2 hits (HRH vs. HH; *p* < 0.05) (Figure 4B). The data suggested that maternal resveratrol was able to decrease increased systolic and mean blood pressure in rats with 2 hits.

### 2.5. Intraperitoneal Glucose Tolerance Test (IPGTT)

**Figure 5:**
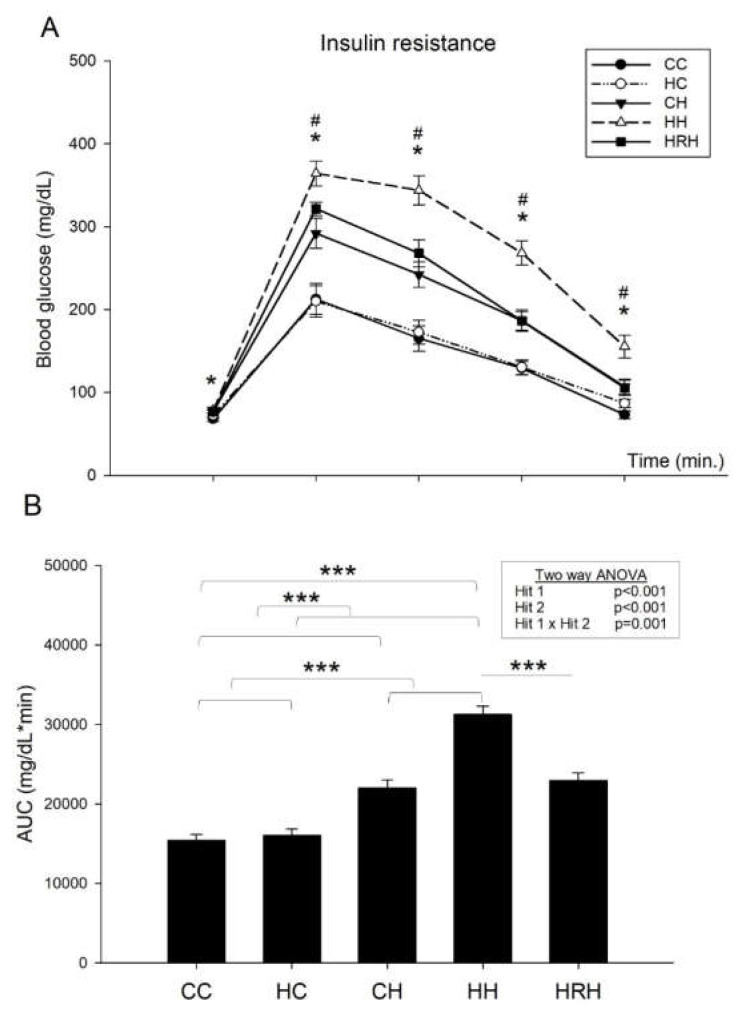
Plasma glucose levels of post-stimulation tests. (**A**) Intraperitoneal glucose tolerance test. * *p* < 0.05 CC vs. HH; # *p* < 0.05 HH vs. HRH (**B**) Glucose area under the curve (AUC). Data were analyzed using two-way ANOVA (maternal diet x post-weaning diet), and the therapeutic effect of resveratrol was evaluated using the Student’s *t*-test. Maternal HFD/obesity treatment in AUC: (F (1,42) = 16.791, *p* < 0.001). Postnatal HFD treatment in AUC: (F (1,42) = 82.249, *p* < 0.001). An interaction effect between maternal HFD/obesity and postnatal HFD in AUC: (F (1,42) = 12.790, *p* = 0.001). *** *p* < 0.001.

The blood glucose levels were measured at the 15, 30, 60, and 120 min marks. In line with our previous report [9], the results indicated that blood glucose levels of rats in the HH group were higher than those of the CC group (*p* < 0.05). In addition, rats received resveratrol had blood glucose levels lowered and showed lower blood glucose level than those of HH group (*p* < 0.05) (Figure 5A). Two-way ANOVA of glucose area under the curve (AUC) showed a significant main effect of maternal HFD/obesity treatment (*p* < 0.001), and a significant main effect of postnatal HFD treatment (*p* < 0.001). There was an interaction effect between maternal HFD/obesity and postnatal HFD in AUC (*p* = 0.001). Student’s *t*-test showed that resveratrol treatment significantly decreased the AUC in animals with 2 hits (HH vs. HRH, *p* < 0.001). These results indicate that the AUC was largest in the HH group than the rest of the three groups (Figure 5B), and treatment with resveratrol reduced the AUC levels to that observed in the controls. Thus, the data shows that combination of maternal HFD/obesity and postnatal HFD results in peripheral insulin resistance, and maternal resveratrol treatment was able to reverse this effect.

### 2.6. Morris Water Maze

**Figure 6:**
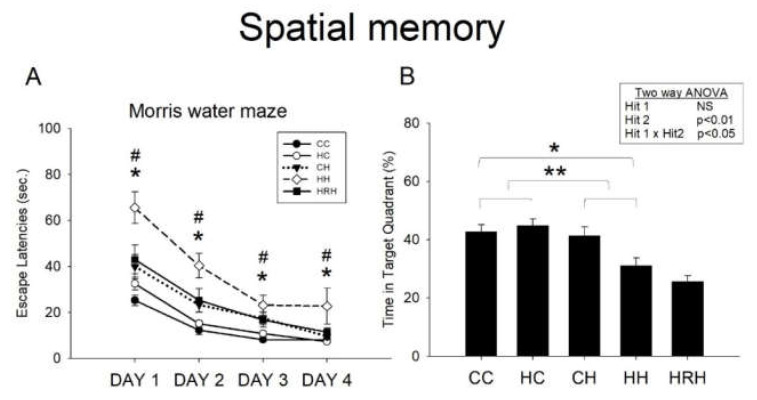
Spatial learning and memory test assessed by the Morris water maze. (**A**) Escape latency to the platform in the Morris water maze (mean ± SEM). Rats in the HH group swam for a longer time to find the submerged platform on all four acquisition days compared to rats in the CC, HC, and CH groups. HRH group rats took less time to reach the platform compared to rats in the HH group. Maternal HFD/obesity on escape latencies: (F (1,36) = 13.949, *p* = 0.001). Postnatal HFD on escape latencies: (F (1,36) = 38.725, *p* < 0.001). An interaction between maternal obesity and postnatal HFD: (F (1,36) = 6.266, *p* < 0.05). (**B**) HH rats on average spent the least amount of time in the target quadrant on day five among the four experimental groups. However, there was no significant difference in the retention time between HH and HRH rats. A two-way ANOVA was used to assess the differences among groups in acquisition phase (repeated measure) and retention. The therapeutic effect of resveratrol was evaluated using the Student’s *t*-test. Postnatal HFD in target quadrant exploration: (F (1,36) = 8.094, *p* < 0.01). An interaction between maternal high-fat/maternal obesity and postnatal HFD in target quadrant exploration: (F (1,36) = 5.147, *p* < 0.05). * *p* < 0.05; ** *p* < 0.01; * *p* < 0.05 vs. CC; # *p* < 0.05 vs. HRH.

Acquisition: The water maze tests revealed that all experimental rats were able to learn how to find the platform, and that there was no significant difference in swim velocity among all the experimental groups at any time (*p* > 0.1). Escape latency improved over time in all five groups as indicated by the significant effect of day of testing on escape latency, indicating that spatial learning occurred (Figure 6A). In line with our previous report [9], we observed a significant main effect of maternal HFD/obesity on escape latencies (*p* = 0.001). In addition, there was a significant main effect of postnatal HFD on escape latencies (*p* < 0.001). There was also an interaction between maternal obesity and postnatal HFD (*p* < 0.05). Post hoc analysis showed that rats in the HH group performed worst compared to rats in the rest of the three groups (*p* < 0.05 in all 4 days) in the Morris water maze test. Further analysis showed that resveratrol treatment significantly decreased escape latencies (HH vs. HRH, *p* < 0.05 in all 4 days).

Retention: In the target zone exploration analysis, two-way ANOVA showed no significant main effect of maternal HFD/obesity, but a significant main effect of postnatal HFD (*p* < 0.01), as well as a significant interaction between maternal high-fat/maternal obesity and postnatal HFD (*p* < 0.05) in target quadrant exploration. Post hoc analysis showed that rats in the CH group performed better in this retention test than rats in the HH group (*p* = 0.008). This result indicates a retention deficit in HH rats. However, the Student’s *t*-test analysis showed that treatment with maternal resveratrol failed to prolong the time spent in the target quadrant (HH vs. HRH, *p* = 0.206) (Figure 6B).

In agreement with our previous report [9], the combination of maternal HFD/obesity and exposure to a postnatal HFD resulted in a significant impairment of acquisition and retention in the Morris water maze test. Furthermore, treatment with maternal resveratrol was able to rescue spatial acquisition deficit in HH group rats.

### 2.7. Biochemistry Parameters

The levels of Aspartate aminotransferase (AST), Alanine aminotransferase (ALT), and total cholesterol were all highest in HH group rats (post hoc, HH vs. CC, CH, and HH, all *p* < 0.001) (Table 1). Student’s *t*-test analysis showed that maternal resveratrol treatment was able to restore AST, ALT, and total cholesterol levels (HH vs. HRH; *p* < 0.001). However, there was no significant difference in triglyceride among the four groups (Table 1).

### 2.8. Protein Levels Involved in Cognition and Insulin Signaling in Dorsal Hippocampus in 4-Month-Old Male Offspring

**Figure 7:**
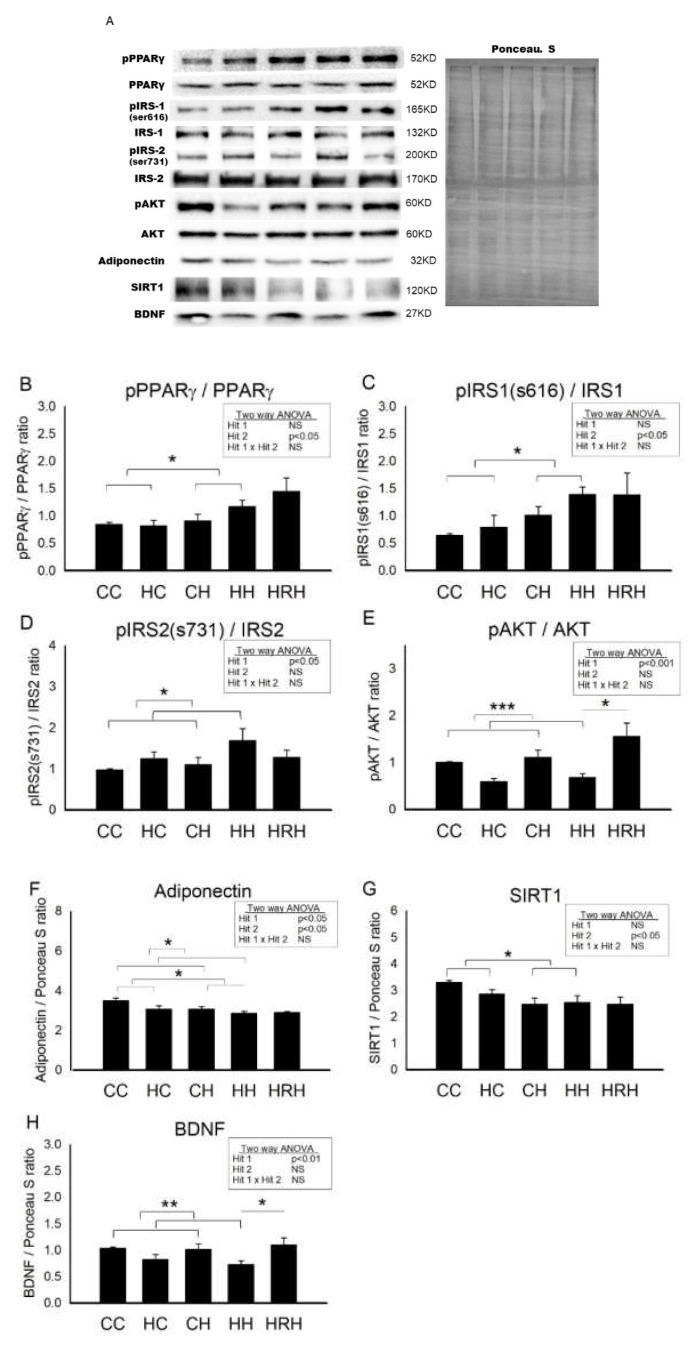
Targeted protein levels in rat dorsal hippocampus at 4 months old. The levels of dorsal hippocampus targeted proteins were detected via Western blotting and normalized using Ponceau S staining. (**A**) Representative band densities are shown. Relative abundance of (**B**) pPPARγ/PPARγ, (**C**) pIRS1 (s616)/IRS1, (**D**) pIRS2 (s731)/IRS2, (**E**) pAKT/AKT, (**F**) adiponectin, (**G**) SIRT1, and (**H**) BDNF were quantified. One-way ANOVA followed by Fisher’s LSD post hoc was used to assess the significant differences among groups and multiple comparisons. pPPARγ/PPARγ: postnatal HFD treatment (F (1,36) = 4.170, *p* < 0.05). pIRS(s616)/IRS1: postnatal HFD treatment (F (1,28) = 6.817, *p* < 0.05). pIRS2 (s731)/IRS2: maternal HFD/obesity treatment (F (1,24) = 5.094, *p* < 0.05). pAKT/AKT: maternal HFD/obesity treatment (F (1,28) = 20.507, *p* < 0.001). Adiponectin: maternal HFD/obesity (F (1,32) = 5.187, *p* < 0.05); postnatal HFD treatment (F (1,32) = 4.941, *p* < 0.05). SIRT1: postnatal HFD treatment (F (1,44) = 8.475, *p* < 0.01). BDNF: maternal HFD/obesity treatment (F (1,44) = 11.145, *p* < 0.01). *n* = 10–12 for each group. * *p* < 0.05; ** *p* < 0.01; *** *p* < 0.001. PPARγ: peroxisome proliferator-activated receptors γ; IRS: insulin receptor substrate; AKT: alpha serine/threonine-protein kinase; SIRT1: sirtuin 1; BDNF: brain-derived neurotrophic factor.

Figure 7 showed the representative blots of proteins involved in cognition and insulin signaling examined in the dorsal hippocampus of 4-month-old offspring. Two-way ANOVA showed a significant main effect for postnatal HFD treatment (*p* < 0.05), but not for maternal HFD/obesity in pPPARγ/PPARγ levels. There was no interaction between maternal HFD/obesity and postnatal HFD. Student’s *t*-test analysis showed that resveratrol treatment could not restore hippocampal pPPARγ levels (HH vs. HRH; *p* > 0.05) (Figure 7B). In order to determine the effect of maternal HFD/obesity and postnatal HFD on central insulin resistance, we assayed the level of pIRS1 in the dorsal hippocampus [28]. Two-way ANOVA showed a main effect of postnatal HFD treatment (*p* < 0.05), indicating an increase in the level of pIRS1 (i.e., decreased IRS-1 downstream signaling), but not for maternal HFD/obesity in pIRS1 levels. There was no interaction between maternal HFD/obesity and postnatal HFD. Resveratrol treatment failed to restore the levels of pIRS1 (HH vs. HRH; *p* > 0.05) (Figure 7C). Two-way ANOVA revealed a main effect of maternal HFD/obesity treatment (*p* < 0.05), but not postnatal HFD on pIRS2 levels in the hippocampus. There was no significant interaction effect between maternal HFD/obesity and postnatal HFD. Resveratrol treatment failed to restore the levels of pIRS2 (HH vs. HRH; *p* > 0.05) (Figure 7D). AKT is the downstream mediator of IRS. Two-way ANOVA revealed a main effect of maternal HFD/obesity treatment (*p* < 0.001), but not postnatal HFD on pAKT/AKT levels in the hippocampus. There was no interaction between maternal HFD/obesity and postnatal HFD. Student’s *t*-test showed that resveratrol treatment could restore hippocampal pAKT/AKT levels (HH vs. HRH; *p* < 0.05) (Figure 7E). Previous studies have linked decreased plasma adiponectin levels with insulin resistance and cognitive dysfunction [29,30,31]. Two-way ANOVA showed a main effect of maternal HFD/obesity (*p* < 0.05) as well as postnatal HFD treatment (*p* < 0.05) on hippocampal adiponectin levels. However, there was no significant interaction effect between maternal HFD/obesity and postnatal HFD (*p* > 0.05). Student’s *t*-test showed that resveratrol treatment was unable to restore hippocampal adiponectin levels in HRH group rats as compared with the levels in HH rats (*p* > 0.05) (Figure 7F). Decreased hippocampal SIRT1 expression is noted in the context of obesity/HFD [9]. Two-way ANOVA showed a main effect of postnatal HFD treatment on levels of SIRT1 (*p* < 0.01) in the hippocampus. However, there was no significant effect of maternal HFD/obesity on the SIRT1 level. There was also no interaction between maternal HFD/obesity and postnatal HFD on the level of hippocampal SIRT1 (*p* > 0.05). Student’s *t*-test showed that resveratrol treatment failed to restore hippocampal SIRT1 level (HH vs. HRH; *p* > 0.05) (Figure 7G). BDNF is critically involved in hippocampal injury in the context of obesity/HFD [9]. Two-way ANOVA showed a main effect of maternal HFD/obesity treatment (*p* < 0.01), but not postnatal HFD on hippocampal BDNF level. There was no interaction between maternal HFD/obesity and postnatal HFD. Student’s *t*-test showed that resveratrol intake was able to restore hippocampal BDNF level in the HRH group (HH vs. HRH; *p* < 0.05) (Figure 7H). Collectively, decreased adiponectin, pAKT, SIRT1, and BDNF were detected in dorsal hippocampus in adult male rat offspring with 2-hit and maternal resveratrol treatment restored hippocampal pAKT and BDNF.

## 3. Discussion

The major findings of this study were (1) maternal HFD/obesity decreased adiponectin, pAKT, SIRT1, and BDNF in rat placenta, fetal brain, and adult male offspring dorsal hippocampus; (2) maternal resveratrol treatment restored adiponectin, pAKT, and BDNF in male fetal brain; (3) maternal resveratrol treatment restored hippocampal pAKT and BDNF in rats with combined maternal HFD and postnatal HFD in adult male offspring dorsal hippocampus; (4) maternal resveratrol treatment reduced increased body weight, peripheral insulin resistance, increased blood pressure, and alleviated cognitive impairment in adult male offspring with combined maternal HFD and postnatal HFD.

AKT signaling pathway interacts with insulin and the mammalian target of rapamycin, and is linked with fetoplacental vascular development [32]. SIRT1 is associated with placental development by controlling extravillous trophoblast invasion and spiral artery remodeling [33]. Adiponectin contributes to the hormonal control of fetal growth and is involved in compromised pregnancy [34]. BDNF plays an autocrine/paracrine role during implantation, placental development, and fetus growth [35]. To maintain an adequate BDNF level is particularly important during early life, when BDNF level and brain plasticity are elevated and diseases have not yet established [36]. We focused on the above mediators to link maternal HFD/obesity and programming.

The placenta is an important organ vital for fetal growth, and its compromised function is associated with abnormal fetus development. In the placenta, maternal obesity leads to higher placenta weight, placenta vascular dysfunction, altered nutrient handling, placenta inflammation, energy modulation, and reduced angiogenesis [37,38].

In a human study, maternal obesity was associated with the downregulation of adiponectin systems in term placenta [39]. In a mice model of maternal HFD, increased PPARγ and decreased SIRT1 were detected in placenta [26]. Increased pAKT in mice placenta from obese dams was reported, suggesting increased maternal circulating insulin [40]. Maternal obesity decreased proBDNF in placentas from male fetuses. In addition, maternal obesity adversely affects BDNF/TRKB signaling in the placenta that is sex-dependent [41]. In this study, we observed that maternal HFD/obesity increased pPPARγ/PPARγ and decreased adiponectin, pAKT, SIRT1, and BDNF in rat placenta while maternal resveratrol treatment was unable to affect the above-mentioned molecules in the placenta.

Fetuses of obese mothers are at increased risks of macrosomia and intrauterine growth retardation [5]. A very limited number of studies have indicated that maternal obesity might alter brain development in the offspring. In a human study, maternal obesity with BMI ≥ 30 affects fetus gene expression at term, implicating aberrant brain development, inflammatory and immune signaling, glucose and lipid homeostasis, and oxidative stress [42]. Stachowiak et al. examined the fetal brain of term fetuses of obese rats fed a HFD. The authors found increased inflammation and oxidative stress in the fetal brains of obese dams, and dysregulation of monoamine neurotransmitter signaling and hypothalamic orexigenic signaling [43]. In a mice study, obese dams affected fetal hippocampal development on embryonic day 17, as shown by the increased proliferation of neural precursors, decreased apoptosis, and by the decreased differentiation of calretinin-positive neurons within the dentate gyrus [44]. Resveratrol can cross the placenta and affect the fetus directly [45]. In this study, we observed that maternal HFD/obesity decreased adiponectin, pAKT, SIRT1, and BDNF in rat fetal brain, and maternal resveratrol treatment was able to restore adiponectin, pAKT, and BDNF in male fetal brain.

Maternal HFD/obesity may program offspring adipose tissue inflammation, adiposity, dysmetabolism, and these programmed changes are exacerbated by a second hit of high-fat diet later in life [9,46]. From the body weight perspective, our data suggest that resveratrol was able to alleviate intrauterine insult induced by maternal obesity/HFD. This might result from resveratrol’s effect on uterus [47]. At ~PND 120, resveratrol was able to alleviate obesity in rats with 2-hit, compatible with the anti-obesity effect of resveratrol [17]. Our rat model might imply an adverse intrauterine insult followed by a rapid postnatal growth catch-up.

Adiponectin, secreted by adipose tissue, is correlated with body mass index and fat mass [48]. Decreased plasma adiponectin levels was reported to be associated with mild cognitive dysfunction [31]. Hippocampal adiponectin level is also known to be associated with cognitive function [49]. AKT is a downstream molecule of IRS and plays a crucial role in insulin signaling and cognition. HFD-induced obesity may impair memory that is mediated by neuroepigenetic dysregulation of SIRT1 within the hippocampus [50]. Resveratrol has substantial effects on SIRT1 levels and deacetylase activity in vitro or in vivo [51]. In this study, we did not detect obvious effect of Sirt 1 in placenta, fetus, or adult offspring following resveratrol intake. Further studies with higher resveratrol dosages and longer treatment duration are suggested. BDNF is involved in the pathogenesis of obesity, type 2 diabetes mellitus, and metabolic syndrome [52,53]. Previous studies have demonstrated that reduction in hippocampal BDNF, resulting from intake of a HFD, may impair learning and memory [54,55,56]. Resveratrol also confers antidepression-like effects possibly via upregulation of hippocampal BDNF in rats [57]. Insulin sensitivity is known to be regulated via serine/threonine phosphorylation of IRS-1, in which phospho-IRS-1-Ser636 has been shown to be associated with desensitization of insulin signaling [28]. Furthermore, insulin receptor signaling is associated with cognitive function [58].

In this study, we found that maternal HFD/obesity decreased adiponectin, pAKT, SIRT1, and BDNF in the adult male offspring dorsal hippocampus. In addition, we found that maternal resveratrol treatment was able to restore hippocampal pAKT and BDNF in rats with combined maternal HFD and postnatal HFD in adult male offspring dorsal hippocampus. We have previously shown that maternal HFD/obesity and postnatal HFD result in metabolic syndrome in adult rat male offspring [9]. Here, we showed that maternal resveratrol treatment was able to reduce increased body weight, peripheral insulin resistance, and increased blood pressure, and alleviated cognitive impairment in adult male offspring with combined maternal HFD and postnatal HFD.

In human studies, resveratrol has been shown to be safe at doses up to 5 g per day [59]. In terms of reproductive toxicity assay, resveratrol 750 mg/kg/day did not result in any unfavorable reproductive effects in an embryo-fetal study [60]. The use of resveratrol in adverse human pregnancies is under investigation [12]. In this work, we found that maternal resveratrol intake was effective in reprogramming adversities of maternal HFD/obesity.

**Figure 8:**
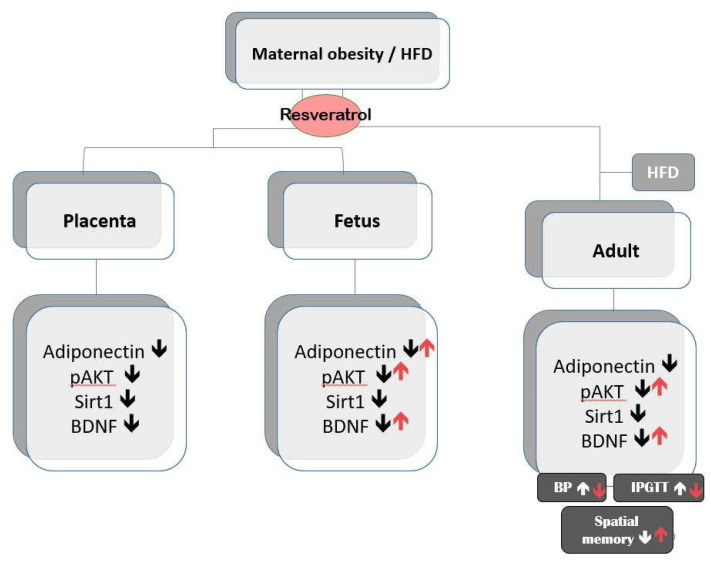
A summary of the effects of maternal resveratrol on the maternal high-fat diet/obesity with or without postnatal high-fat diet. ↑ indicates up-regulation; ↓ indicates down-regulation.

The maternal programming effects begin from the gestation period and encompass the early postnatal period, when offspring are exposed to the combined effects of milk composition and maternal behavior. The peripheral metabolic changes exhibited by maternal HFD-exposed offspring may also contribute to offspring adverse development. Mounting evidence supports that both maternal programming and offspring metabolic impairment in contributing to offspring metabolic and cognitive impairment. Our study and others [61] provide support that maternal environment can have long-lasting consequences on offspring metabolic and cognition development. Alongside with other studies [19], our results indicate that reprogramming in the maternal stage with resveratrol is a possible avenue to avoid detrimental effects of maternal HFD/obesity. Figure 8 summarizes the main findings of maternal resveratrol in the context of maternal HFD/obesity with or without postnatal HFD.

This study has a few limitations. First, this study used only male animals. Therefore, future studies are needed to elucidate the gender impact in this context. Second, the pups from maternal obesity/high-fat diet were lighter in birth weight as compared with control. However, in clinical practice neonates are always heavier from obese mothers. Finally, our resveratrol treatment timeline did not include lactation period. Since rats were born prematurely as compared with human, future studies to administer resveratrol including both gestation and lactation period are needed. Then, the implication of resveratrol in reprogramming adverse long-term effects in compromised pregnancy can be more generalized to clinical situation.

## 4. Materials and Methods

### 4.1. Animals and Treatment

Animal experiments were carried out at the Animal Experimental Center of Kaohsiung Chang Gung Memorial Hospital. This work was approved by the Institutional Animal Care and Use Committee of the Kaohsiung Chang Gung Memorial Hospital (Approval No. 2017031307; Valid period: 01/07/2017~30/06/2019). This experiment was carried out in accordance with the recommendations of the Guide for the Care and Use of Laboratory Animals of the National Institutes of Health. Virgin Sprague-Dawley (SD) rats (BioLASCO Taiwan Co., Ltd., Taipei, Taiwan) were housed and maintained in our animal care center accredited by the Association for Assessment and Accreditation of Laboratory Animal Care International. The rats were exposed to a 12 h light/12 h normal light-dark cycle. Female SD rats were caged with male rats until mating was visually confirmed by vaginal plug.

**Figure 9:**
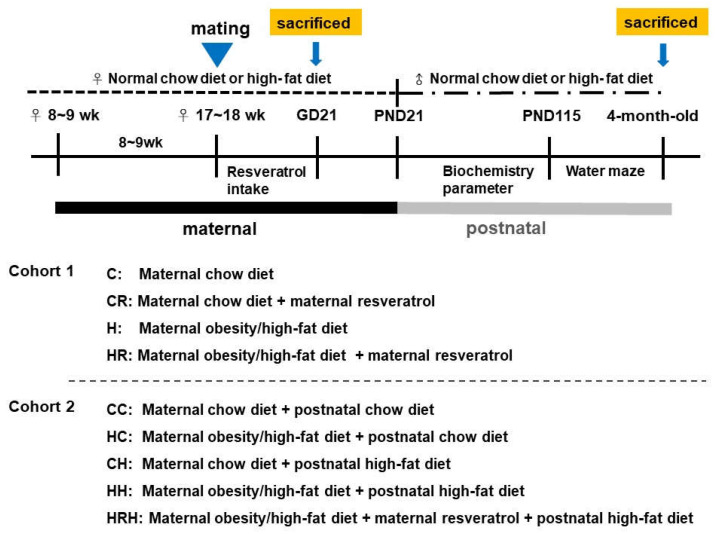
The experimental design and timeline. Approximately 8–9-week-old female rats were fed a chow diet or HFD for 8 weeks. Placenta and fetal brains were collected for cohort 1 study. After mating and delivery of pups, male baby rats were weaned and fed a chow diet or a HFD from PND 21 onwards for cohort 2 study. Cohort 1 included four experimental groups (*n* = 12–14 per group). C: maternal rat chow; CR: maternal rat chow diet + maternal resveratrol; H: maternal HFD/obesity; HR: maternal HFD/obesity + maternal resveratrol. Cohort 2 included five experimental groups (*n* = 12–14 per group). CC: maternal rat chow diet + postnatal chow diet; HC: maternal HFD/obesity + postnatal chow diet; CH: maternal rat chow diet + postnatal HFD; HH: maternal/HFD/obesity + postnatal HFD; HRH: maternal HFD/obesity + maternal resveratrol + postnatal HFD.

Experimental design was as shown in Figure 9. Female rats were weight-matched and assigned to receive either a normal diet with regular rat chow (ND; Fwusow Taiwan Co., Ltd., Taichung, Taiwan; 52% carbohydrates, 23.5% protein, 4.5% fat, 10% ash, and 8% fiber) or high-fat hypercaloric diet (HF; D12331, Research Diets, Inc., New Brunswick, NJ, USA; 58% fat) ad libitum for 8 weeks before mating, and throughout gestation and lactation periods. In cohort 1 with four experimental group rats, maternal HFD or rat chow diet with/without resveratrol [62] were used to evaluate the effects of maternal resveratrol intake, 50 mg/L was put in drinking water during the pregnancy as we previously reported [62], in the context of maternal HFD/obesity. Four experimental groups were designated C, CR, H, and HR groups. In cohort 2, litters were culled to eight pups after birth, with equal numbers of each sex to standardize the quantity of milk received and maternal pup care. Only male fetuses and adult offspring were collected because males experienced a greater insult from maternal obesity than females [46]. A maximum of three male offspring were taken from each litter to avoid any litter effects. The offspring of both sexes were weaned at PND 21 onto either the normal diet or HFD ad libitum up to ~4 months of age, as we previously reported [9]. For evaluation of long-term effects, five experimental groups (*n* = 10–14 per group) were allocated: CC group, HC group, CH group, and HH group [9]. In addition, a treatment group with resveratrol 50 mg/L in drinking water during the pregnancy on maternal HFD/postnatal HFD was generated for comparison (HRH). Experiment 1 was designed to assess the acute effects of maternal resveratrol in dams with maternal HFD/obesity and their placenta and male fetuses. Experiment 2 was designed to assess the possible reprogramming effects of maternal resveratrol in male offspring of dam with maternal HFD/obesity with/without a postnatal HFD. Figure 9 showed the experimental design and timeline used in this study.

### 4.2. Tissue Collection and Blood Sampling

In cohort 1, dams were euthanized using 25 mg/kg Zoletil and 23 mg/kg Xylazine. Placenta and fetal brain were collected on gestational day (GD) ~21 by Cesarean section and stored at −80 °C. Only male fetuses, determined by tail tip Sry genotyping, were saved for later analyses. For comparison with adult male offspring, only male fetal brains were used for analyses. In cohort 2, at the age of 4 months, the animals were weighed, and then euthanized using 25 mg/kg Zoletil and 23 mg/kg Xylazine. Plasma and dorsal hippocampus were collected (*n* = 10–14 per group).

### 4.3. Blood Pressure

Blood pressure was measured in conscious rats at ~4 months of age using an indirect tail-cuff method (BP-2000, Visitech Systems, Inc., Apex, NC, USA) according to the manufacturer’s instruction, as we previously described [63,64].

### 4.4. Intraperitoneally Injected Glucose Tolerance Test (IPGTT)

On PND ~110, after an 8-h fast blood samples were collected at 5 time points: before injection and at 15, 30, 60, and 120 min after the intraperitoneal injection of glucose (2 g/kg). Plasma glucose levels were measured using a commercial kit, while serum insulin levels were measured using an immunosorbent assay (Crystal Chem Inc., Downers Grove, IL, USA), as we reported previously [65].

### 4.5. Morris Water Maze: Spatial Memory

The Morris water-maze test was conducted to assess spatial learning and memory in adult male offspring [66]. Briefly, on PND ~115, each rat was habituated to the testing environment. Between PND ~116 and 119, the rats were subjected to six trials per day to locate a submerged platform. The starting position was different with each test trial and the visual cues were kept the same. This period was considered the acquisition phase. On PND ~120, retention of memory was tested with the platform removed.

### 4.6. Biochemistry

After completion of behavioral study, triglyceride, cholesterol, and liver transaminase in plasma were measured using an automated clinical chemistry analysis machine (FUJI DRI-CHEM NX500, Japan).

### 4.7. Western Blotting

Western blot analysis was performed as described previously [67]. Total protein extracts from dorsal hippocampus were lysed in ice-cold RIPA buffer with protease and phosphatase inhibitor cocktails (Roche, Indianapolis, IN, USA). Following centrifugation, protein concentrations in supernatants were determined using the commercial kit (Bio-Rad, Hercules, CA, USA). Briefly, proteins of the dorsal hippocampus were isolated, separated by electrophoresis, transferred to a poly(vinylidene fluoride) membrane, and probed with the investigated primary antibodies: IL-6, IL-1β, PPARγ, pPPARγ, adiponectin, AKT, pAKT, IRS1, pIRS1, IRS2, pIRS2, SIRT1, and BDNF (Table 2). All blots were stained with Ponceau S that serves as an internal standard, as we previously described [66,68]. Bands of interest were visualized using electrochemiluminescence reagents (PerkinElmer, Waltham, MA, USA), and quantified using densitometry (Quantity One Analysis software, Bio-Rad), as the integrated optical density (IOD) after subtraction of background. The IOD was baseline that adjusted from Ponceau S-staining to correct possibly variations in total protein loading [66,69,70]. The expression of protein was represented as IOD/Std.

### 4.8. Statistical Analysis

For placenta and fetus brain, the results were analyzed using one-way analysis of variance (ANOVA), then followed by Fisher’s least-significant-difference (LSD) Post hoc test. For adult offspring, the results were analyzed using two-way ANOVA (maternal diet × postweaning diet) followed by Fisher’s LSD post hoc tests if there was a significant interaction. For all the variables measured, outliers that lay 1.5 interquartile ranges (IQRs) below the first quartile or 1.5 IQRs above the third quartile were removed from further analysis. If there was a synergistic effect of maternal obesity and postnatal HFD, then the therapeutic effect of resveratrol was evaluated using an unpaired Student’s *t*-test. Results from the Morris water maze acquisition phase and IPGTT were subjected to two-way ANOVA (maternal diet × postweaning diet) with repeated measures (time). All analyses were performed using Statistical Package for the Social Sciences (SPSS) software (version 15). Values were expressed as mean ± SEM. A value of *p* < 0.05 was considered statistically significant.

## 5. Conclusions

Maternal resveratrol intake protects the male fetal brain in the context of maternal HFD/obesity in an in vivo rat model. Maternal resveratrol intake can reduce metabolic abnormalities and alleviate cognitive deficit in adult male offspring with combined maternal and lactational HFD, and postnatal HFD. Maternal resveratrol intake may have translational use in the context of maternal HFD/obesity.

## Figures and Tables

**Table 1:** Biochemistry of adult male offspring in rats with maternal high-fat diet (HFD)/obesity and postnatal high-fat diet.

Groups	CC	HC	CH	HH	HRH	Two-Way ANOVA (*p* Value)		
Rat numbers	*n* = 14	*n* = 13	*n* = 16	*n* = 14	*n* = 14	Hit 1	Hit 2	Hit 1 × Hit 2
**AST (U/L)**	99.43 ± 10.24	98.23 ± 7.6	226.88 ± 45.58	431.93 ± 80.82	168.50 ± 60.91^###^	<0.05	<0.0001	0.036
**ALT (U/L)**	24.29 ± 1.55	29.77 ± 1.03	153.75 ± 48.57	259.43 ± 52.55	80.29 ± 32.35 ^###^	NS	<0.0001	NS
**Cholesterol** **(mg/dL)**	65.00 ± 3.66	73.00 ± 4.07	68.25 ± 5.78	92.14 ± 5.80	54.14 ± 2.95 ^###^	<0.05	<0.05	NS
**Triglyceride** **(mg/dL)**	77.00 ± 9.87	79.63 ± 8.23	82.88 ± 10.81	74.63 ± 4.77	78.13 ± 7.48	NS	<0.05	NS

AST: Aspartate aminotransferase; ALT: Alanine aminotransferase. ### *p* < 0.001 vs. HH

**Table 2:** Primary and secondary antibodies used for Western blot.

Target	Company	Catalog	Dilution Factor
IL-6 (interleukin 6)	Abcam	ab9324	1:1000
IL-1β (interleukin 1 beta)	Abcam	ab9722	1:1000
Adiponectin	Abcam	ab62551	1:1000
BDNF	Santa cruz	Sc-546	1:1000
pPPARγ (phosphoryl peroxisome proliferator-activated receptors γ)	Bioss	bs-3737R	1:1000
PPARγ (peroxisome proliferator-activated receptors γ)	Santa cruz	7196	1:1000
pAKT (phosphoryl alpha serine/threonine-protein kinase)	Cell signaling	#9271	1:1000
AKT (alpha serine/threonine-protein kinase)	Cell signaling	#9272	1:1000
pIRS1 (phosphoryl insulin receptor substrate 1)	Millipore	#07-843	1:1000
IRS1 (insulin receptor substrate 1)	Cell signaling	#2382	1:1000
pIRS2 (phosphoryl insulin receptor substrate 2)	abcam	ab3690	1:1000
IRS2 (insulin receptor substrate 2)	Abclonal	A7945	1:1000
SIRT1 (sirtuin 1)	Abcam	ab104833	1:1000
